# Effects of the COVID-19 pandemic on the physical activity and screen time habits of children aged 11–13 years in Sweden

**DOI:** 10.3389/fpubh.2023.1241938

**Published:** 2023-08-09

**Authors:** Sara Berggren, Gerd Almquist-Tangen, Olivia Wolfbrandt, Josefine Roswall

**Affiliations:** ^1^Department of Pediatrics, Sahlgrenska Academy, Institute of Clinical Sciences, University of Gothenburg, Gothenburg, Sweden; ^2^Halland Health Center Hyltebruk, Hyltebruk, Sweden; ^3^Child Health Care Unit, Halmstad, Sweden; ^4^Department of Pediatrics, Halland Hospital Halmstad, Halmstad, Sweden

**Keywords:** physical activity, exercise, screen time, lifestyle habits, COVID-19, children, adolescents, Sweden

## Abstract

**Introduction:**

Physical activity (PA), exercise, sedentary behavior and screen time are lifestyle factors that have been shown to significantly impact child health in different ways. These lifestyle factors were affected to different degrees by global restrictions during the COVID-19 pandemic. We investigated PA and screen time in a cohort of Swedish children in both 2019 and 2021, before and during the pandemic.

**Method:**

Adolescents born in 2008 in Halland, Sweden, and included in a previous longitudinal birth cohort study were invited to take part in follow-up questionnaires about PA, screen time and COVID-19. A total of 1041 children aged 11 (in 2019) and 13 years (in 2021) replied and 777 of them answered on both occasions.

**Results:**

Most children (42.1%) reported that their leisure time PA was unchanged from 2019 to 2021. Compared to unchanged PA 33.9% exercised more often (*p* = 0.011) and 23.9% exercised less (*p* < 0.001), both differences statistically significant. Roughly, 43.2% of boys and 34.9% of girls in 2021 exercised so that they became breathless or broke a sweat at least 4 times a week not counting physical education in school, corresponding figures for 2019 were 38.2% for boys and 35.2% for girls. The majority of children were able to continue attending leisure time sports clubs during the pandemic, but participation decreased from 88.3% to 76.3% from 11 to 13 years of age. Most reported that sports club routines changed during the pandemic, but only 40.9% reported fewer practice opportunities. Attending a sports club gave greater protection against loss of PA during the pandemic than not belonging to one (41.0% vs. 23.2%, *p* < 0.001). The majority (71.1%) of children spent more time on screens in 2021 than 2019, with a mean increase of 9.4 h (95% CI 8.6 to 10.2 h) from 20.7 to 30.1 hours per week (*p* < 0.001) during the study.

**Conclusions:**

Swedish children largely maintained their levels of PA during the pandemic at 13 years of age and these were possibly safeguarded by the comparably mild pandemic restrictions in Sweden in 2021. However, they did increase their screen time between 11 and 13 years of age.

## Introduction

Universal child health guidelines states that children should engage in physical activity (PA), eat fruit and vegetables, sleep enough and limit sedentary time and sugary drinks. In the 2020 guidelines, The World Health Organization (WHO) recommends that children and adolescents aged 5–17 should engage in an average of 60 min/day, of moderate-to-vigorous intensity, mostly aerobic, physical activity (MVPA), with moderate physical activity usually being a 5 or above on a scale of 0–10. Vigorous-intensity aerobic activities, as well as those that strengthen muscle and bone should be incorporated at least 3 days a week. Further on, it is strongly recommended that children and adolescents should limit the amount of time spent being sedentary, particularly the amount of recreational screen time (1). Although the WHO has not set an established time limit for recreational screen time, consensus documents have suggested a maximum of 2 h a day (2, 3) as levels above this have been associated with health risks (4–6). According to this consensus document (2), recreational screentime includes all discretionary and leisure-time screen time done while sedentary and typically includes television viewing, video-games use and computer use. One of the reasons for setting specific time limits is to prevent childhood obesity (7) and cardiovascular and metabolic diseases in adulthood (8). In 2016, Guthold et al. (9) reported that globally 81% of children aged 11–17 did not meet the PA recommendations and other studies have shown that PA reduces with age (10, 11). Further on, for children and adolescents' physical activity has an important role in improving motor and cognitive development (3). On the other hand, the increasing and widespread recreational use of screens by children may increase sedentary time and reduce the time available for PA (10). In addition, increased screen time has been associated with the increased consumption of soft drinks and snacks (11, 12) and decreased intake of fruit and vegetables (13). It has also been identified as an independent risk factor for poor health (5, 6). Physical activity and screen time are two of the lifestyle factors that, together with genetic predisposition, can affect childhood health and the development of overweight and obesity in children (14).

Socioeconomic status affects lifestyle and studies have shown that children from families with lower socioeconomic status are more affected by societal crises and generally have higher screen time and lower PA levels than more privileged children (15, 16). As an example, attending sports clubs is a costly leisure activity in Sweden that is easily down prioritized when the family income lowers. Fear and alienation of catching disease in a society may also spread in an uneven way. Fear of having to stay at home from work due to an infection may keep a family inside reducing PA and increasing screen time. It has been reported that COVID-19 restrictions negatively influenced PA and screen time for children in a number of countries (17–20). In China, which was one of the countries that went into complete lockdown, PA more than halved and screen time increased considerably (18). This adversely affected the development of obesity in Chinese children (21). The same lifestyle patterns were seen in Italy (22), Croatia (23) and Canada (24). A study of 10 different European countries, which had different restrictions, reported that PA habits were roughly the same before and during the pandemic (19). However, the picture was different in Germany (25) and Belgium (26), which had milder restrictions for children than others. Schools were closed and social distancing rules were strict, but outdoor activities were allowed for children and this may have led to increased PA of low to moderate intensity. This was presumably due to children spending more time outdoors and a strong medical focus on healthy behavior (25, 26). Despite that, all those studies reported increased screen time and stated that most children did not meet the general recommendations of < 2 h of screen time per day during the pandemic (2).

During the COVID-19 pandemic, Swedish individuals aged fifteen or younger did not face the strict restrictions imposed by many other countries, but were still affected by some social distancing measures in 2021. For instance, schools were open for this age group and organized physical activities for children continued during evenings and weekends in 2021, but general social distancing and contact restrictions applied (27). This meant that fewer participants were involved, activities were held outdoors if possible and most sports competitions were canceled. These mild restrictions were criticized, as they risked transmission, but the aim was to safeguard this age group's education and provide them with opportunities for PA (27). It is interesting to study lifestyle habits in different countries during the pandemic due to the different restrictions that were applied. In some cases, these restrictions changed lifestyle factors, but not always in the ways that were intended or predicted.

The aim of this study was to describe PA and screen time habits for a cohort of Swedish children aged 11 and 13, before and during the COVID-19 pandemic, and relate the results to other countries where other restrictions applied.

## Materials and methods

### Study design

**Figure 1:**
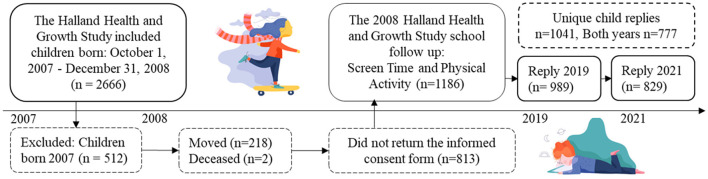
Flowchart of the study population. This study was part of the follow up for the Halland Health and Growth Study, a longitudinal, population-based, birth cohort study that recruited children born between October 1, 2007, and December 31, 2008. Total births in the county (*n* = 3,680) in 2008.

This study formed part of the larger Halland Health and Growth Study (H^2^GS) (28), an ongoing population-based birth cohort that is following 2666 children born in Halland, southwest Sweden, between October 1 2007 and December 31 2008. No exclusion criteria were applied, but parents had to understand Swedish well enough to provide informed consent and understand the questionnaires, which were only available in Swedish. For this study, 1,934 were eligible and asked to participate in the follow-up H^2^GS Goes to School study (Figure 1). In total, 1,186 (59.3%) returned the informed consent form and questionnaires regarding self-estimated screen time and PA were sent out digitally.

### Participants

Sociodemographic data, collected at birth, are presented in Table 1. The children who took part differed from the drop-outs with regards to a number of maternal factors: age at childbirth, pre-pregnancy body mass index (BMI), descent and educational level. However, there were no differences in child-related factors, namely weight, gestational age at birth and sex, as shown in Table 1. The same 1,934 children were given the opportunity to participate in 2019 and 2021 and the 1,041 who did take part in one or both years represented a response rate of 53.9% and covered 28.3% of all the children who were born in Halland 2008 and still living in the area. The response rate at the age of 11 (in 2019) was 989 children (51.2%), which covered 26.9% of the population, and at the age of 13 (in 2021) it was 829 (42.9%), covering 22.5% of the children of that age. Of these 829 children replying in 2021, 777 took part in both years.

**Table 1:** Background data for the study population in comparison with drop outs, as well as data for the original cohort of the 2,154 children born in 2008.

	**Study population**		**No follow up**			**Original cohort in 2008**	
	**Mean**	***n***=	**Mean**	***n***=	*p* ^*^	**Mean**	*N*^a^=
Birth weight (g)	3,526	1,040	3,525	1,111	0.968	3,526	2,151
Mothers' age at childbirth (years)	31.7	1,041	30.2	1,111	< 0.001	30.9	2,152
Mothers' pre-pregnancy body mass index (kg/m^2^)	23.8	1,036	24.5	1,104	< 0.001	24.2	2,140
	**% (** ***n*** **)**	***n***=	**% (** ***n*** **)**	***n***=	***p***	**% (** ***n*** **)**	***n***=
Fullterm (% born between 37 and 41weeks)	87.5 (911)	1,039	87.8 (975)	1,111	0.956	87.7 (1,886)	2,150
Child's sex (boys)	49.5 (515)	1,041	51.4 (571)	1,093	0.372	50.5 (1,086)	2,152
Mothers' heritage (born in Sweden)	90.6 (943)	1,024	82.9 (921)	1,076	< 0.001	86.6 (1,864)	2,100
Mothers' educational level (>12years)	63.5 (661)	1,031	45.3 (503)	1,093	< 0.001	54.1 (1,164)	2,124

^*^The independent sample *t*-test was used for mean comparisons and the chi-square test was used to compare frequencies.

^a^The number of children varied due to missing data.

### Procedures

Questionnaires were emailed via the parents in May 2019 and sent directly to the children in September 2021. We used virtually identical questions in both years, but added questions about COVID-19 in 2021. Both questionnaires were primarily aimed at the children. The 2019 questionnaire also contained some questions for the parents. Three reminders were sent out. Questionnaires about PA, exercise habits and screen time habits were answered in Swedish and questions are detailed in Supplementary material 1. PA was defined as activity that caused the subjects to become breathless or break a sweat. There was no question defining the amount of time spent physically active and no question that may be directly translated into a scale of 0–10. With this limitation in mind, subjects who said that they exercised at least 4–5 times after school, or at the weekends, were considered to be sufficiently physically active. This because, organized physical activities generally lasts 45–90 min and mandatory physical education at school was offered 60–90 min twice a week during the whole study period. Screen time was defined as the time they spent watching television, on their phones, tablets and computers. Weekdays were defined as Monday to Friday and weekends as Saturdays and Sundays.

### Living conditions and restrictions in Sweden during the COVID-19 pandemic

During the pandemic, adults were advised to work from home if possible, but in 2019 and 2021 all children aged 11 and 13 still attended school in person, with some minor restrictions during the pandemic. Physical education and school breaks were outdoors. Social distancing applied to everyone independent on age and involved restrictions on the number of people allowed in the same room. Adults and children were recommended to socialize outdoors and keep their distance from other people. During the pandemic, the rule was to stay at home if you had any symptoms that suggested that you had the virus and this resulted in high numbers of students and teachers taking sick leave. Schools were allowed to offer socially distanced education from January 2021 to May 2021 for those aged 13–15 years, in addition to regular on-site schooling. In order to reduce the number of children in schools at any one time, so that social distancing could be put in place, some spent half their time at home and the other half in the classroom. However, they were able to go into the school to collect lunch packs or homework and ask questions. These measures were not very common, but they could have affected some of our study cohort in early spring 2021. Physical activity was allowed outdoors or with restrictions on the number of people allowed inside. National restrictions may be studied in detail on the Public Health Agency of Sweden's website and these are available in English (27).

### Data analysis

Descriptive data are reported as numbers and percentages. The statistical analyses were performed using SPSS statistics for Windows, version 29.0 (IBM Corp, New York, USA) and the significance level was set at *p* < 0.05. The paired *t*-test was used for mean differences for normally distributed variables. Differences in the incidence between unrelated groups were analyzed with the chi-square test and McNemar test for paired, longitudinal data. We also studied potential differences in the frequency of reported screen time and PA over time, namely increased, decreased and unchanged, using Pearson chi-square of goodness-of-fit and *post-hoc* pairwise chi-square analysis. Effect sizes for screen time and PA were analyzed with Cramers V and interpreted as < 0.1 for negligible, < 0.3 for small and >0.5 for large. Relative risks were calculated for changes in PA for a better understanding of the effect sizes. Maternal educational level was used as a proxy for socioeconomic status.

## Results

### Physical activity before and during the pandemic

In 2019, 36.7% of the 891 children aged 11 who answered the PA question reported exercising at least 4–5 times a week after school or at the weekends (Table 2). In 2021, at the age of 13, the corresponding figures were 39.0% of 828 children. There was a significant, increase in PA between 2019 and 2021 when we analyzed the data for the 731 children who answered this question both years with their response 2 years earlier (Table 2). Between 2019 and 2021, the frequency of leisure time PA was unchanged for 42.1%, increased for 33.9% and decreased for 23.9%. A chi-square test of goodness-of-fit showed that PA was not equally distributed in the 731 children, (x^2^ = 36.4, *p* < 0.001), with a small effect size (Cramers V = 0.16). The *post-hoc* analysis for the pairwise comparison of PA changes showed that all three proportions were significantly different from each other (*p* < 0.001, *p* < 0.001 and *p* = 0.011 respectively). Most children did not change the frequency of PA, but increases were more common than decreases. The relative risk for increased PA vs. decreased PA was 1.17, for unchanged vs. decreased it was 1.27 and for unchanged vs. increased it was 1.11 (data not shown).

**Table 2:** Differences in life style habits for 11-year olds in 2019 (*n* = 989) and for 13-year olds in 2021 (*n* = 829) as well as differences based on sex or maternal educational level as a socioeconomic marker.

			**Yes**			
**Lifestyle habits for children replying both 2019 and 2021**, ***n*** = **777**	**Total replies**, ***n***		**2019** ***n*** **(%)**	**2021** ***n*** **(%)**	**p** ^a^	**Effect size** ^b^
Sufficient PA^c^	731		255 (34.9)	292 (39.9)	**0.017**	0.341
Reported no PA	731		32 (4.4)	53 (7.3)	**0.009**	0.275
Active in a sports club	714		631 (88.4)	550 (77.0)	**< 0.001**	0.425
Active school breaks^e^	726		202 (27.8)	68 (9.4)	**< 0.001**	0.064
Low ST (0–14 h/w)	724		106 (14.6)	23 (3.2)	**< 0.001**	0.215
High ST (>35 h/w)	707		40 (5.7)	195 (27.6)	**< 0.001**	0.150
			**Yes**			
**Lifestyle habits 2019** ***n*** = **989**^d^**, 2021** ***n*** = **829**	**Total replies**, ***n*** **(Boys n)**	**Overall** ***n*** **(%)**	**Boys** ***n*** **(%)**	**Girls** ***n*** **(%)**	*p* ^a^	**Effect size** ^b^
Sufficient PA^c^ 2019	891 (434)	327 (36.7)	166 (38.2)	161 (35.2)	0.350	0.031
Sufficient PA^c^ 2021	828 (412)	323 (39.0)	178 (43.2)	145 (34.9)	**0.014**	0.086
Reported no PA 2019	891 (434)	452 (5.1)	24 (5.5)	21 (4.6)	0.524	0.021
Reported no PA 2021	828 (412)	642 (7.7)	29 (7.0)	35 (8.4)	0.459	0.026
Active in a sports club 2019	895 (440)	790 (88.3)	376 (85.5)	414 (91.0)	**< 0.001**	0.086
Active in a sports club 2021	827 (412)	631 (76.3)	311 (75.5)	320 (77.1)	**< 0.001**	0.019
Active school breaks 2019^e^	891 (434)	258 (29.0)	177 (40.8)	81 (17.7)	**< 0.001**	0.254
Active school breaks 2021	823 (409)	774 (9.4)	50 (12.2)	27 (6.5)	**0.005**	0.098
Low (0–14 h/w) ST 2019	882 (431)	128 (14.5)	64 (14.8)	64 (14.2)	0.781	0.009
Low (0–14 h/w) ST 2021	827 (410)	24 (2.9)	12 (2.9)	12 (2.9)	0.966	0.001
ST >35 h/w 2019	875 (427)	47 (5.4)	28 (6.6)	19 (4.2)	0.129	0.051
ST >35 h/w 2021	809 (397)	225 (27.8)	113 (28.5)	112 (27.2)	0.685	0.014
			**Yes**			
**Lifestyle habits 2019** ***n*** = **989, 2021** ***n*** = **829**	**Total replies**, ***n*** **(Children with low maternal educational level**^f^ ***n*****)**		**Low maternal educational level** ***n*** **(%)**	**High maternal educational level** ***n*** **(%)**	*p* ^a^	**Effect size** ^b^
Sufficient PA^c^ 2019	883 (312)		104 (33.3)	219 (38.4)	0.139	0.050
Sufficient PA^c^ 2021	821 (282)		88 (31.2)	231 (42.9)	**0.001**	0.114
Reported no PA 2019	883 (312)		20 (6.4)	25 (4.4)	0.189	0.044
Reported no PA 2021	821 (282)		32 (11.3)	32 (5.9)	**0.006**	0.096
Active in a sports club 2019	890 (310)		254 (81.9)	531 (91.6)	**< 0.001**	0.142
Active in a sports club 2021	820 (280)		188 (67.1)	436 (80.7)	**< 0.001**	0.151
Active school breaks^e^ 2019	883 (312)		92 (29.5)	165 (28.9)	0.854	0.006
Active school breaks^e^ 2021	816 (282)		29 (10.3)	48 (9.0)	0.547	0.021
Low (0–14 h/w) ST 2019	875 (310)		39 (12.6)	88 (15.6)	0.229	0.041
Low (0–14 h/w) ST 2021	820 (280)		12 (4.3)	11 (2.0)	0.064	0.065
ST >35 h/w 2019	868 (307)		22 (7.2)	25 (4.5)	0.092	0.057
ST >35 h/w 2021	802 (274)		82 (29.9)	143 (27.1)	0.395	0.030

^a^Comparison of frequencies were performed using the McNemar test for comparison of paired data from 2019 to 2021, and the chi-square test for comparison between sexes and maternal educational level, *p* < 0.05 were considered statistically significant and marked in bold; ^b^Effect size as calculated by Cramers V and interpreted as 0.5 to 0.3 moderate, < 0.3 small, < 0.1 negligible; ^c^Children that reported that they became breathless or broke a sweat at least 4–5 times/weekly in addition to mandatory physical education twice a week; ^d^In 2019, answers for 989 children were obtained, however one of the questions about being a member in a sports club were directed toward the parent, this question was answered by 895 parents of whom 98 children did not answer the other questions directed toward the child. The other questions in 2019 were answered by 891-875 children; ^e^Active school breaks: children answered “often or always” as compared to “sometimes, rarely or never;” ^f^Low educational level ≤ 12y of school, high educational level >12 years of school; PA, Physical Activity; ST, Screen Time.

Table 2 shows that there was a significant difference between the sexes at 13 years of age with regard to the percentages who according to our definition exercised sufficiently: 43.2% of the boys and 34.9% of the girls (*p* = 0.014). The same difference was not evident at 11 years of age. Reports of always or often being physically active during school breaks declined with age and was significantly lower in girls, 40.8% of boys vs. 17.7 % of girls at 11 years of age which declined to 12.2% of boys vs. 6.5% of girls at 13 years of age, *p* < 0.001 respective *p* = 0.005. The relationship between socioeconomic status, measured as maternal educational level, and PA increased during the COVID-19 pandemic. Fewer children exercised at least 4–5 times a week on their leisure time if their mother had a lower, rather than higher, educational level. More children in lower socioeconomic groups reported no exercise at all or that they were not active in sports clubs (Table 2).

About a fourth (27.6%) of the 823 who replied in 2021 said they had been less physically active as a result of the COVID-19 pandemic: 28.3% of boys and 26.9% of girls, data not shown.

Table 2 also shows that the overall percentage of children who were active in sports clubs decreased from 88.3% to 76.3% between 11 and 13 years of age (*p* < 0.001). The vast majority (97.9%) of the 631 children who were active in sports clubs in 2021 said that they were able to continue their activities in some way during the pandemic. However, 63.5% reported that the way they practiced their sport had changed and 40.9% had fewer practice opportunities, due to canceled practice sessions and sports competitions. Other reasons included being outdoors, instead of inside, or practicing with fewer participants. Being a sports club member seemed to protect children against loss of PA in 2021, according to the replies from 822 respondents: 76.5% remained active in sports clubs and 23.2% said that they had been less physically active in 2021 due to COVID-19. This answer was independent of whether they had reported a change in their frequency of PA between the years. In comparison, 41.0% of the 196 children who were not members of sports clubs reported less exercise during the year with COVID-19 (McNemar *p* < 0.001, Cramers V 0.178 small effect size, data not shown).

### Screen time before and during the pandemic

**Figure 2:**
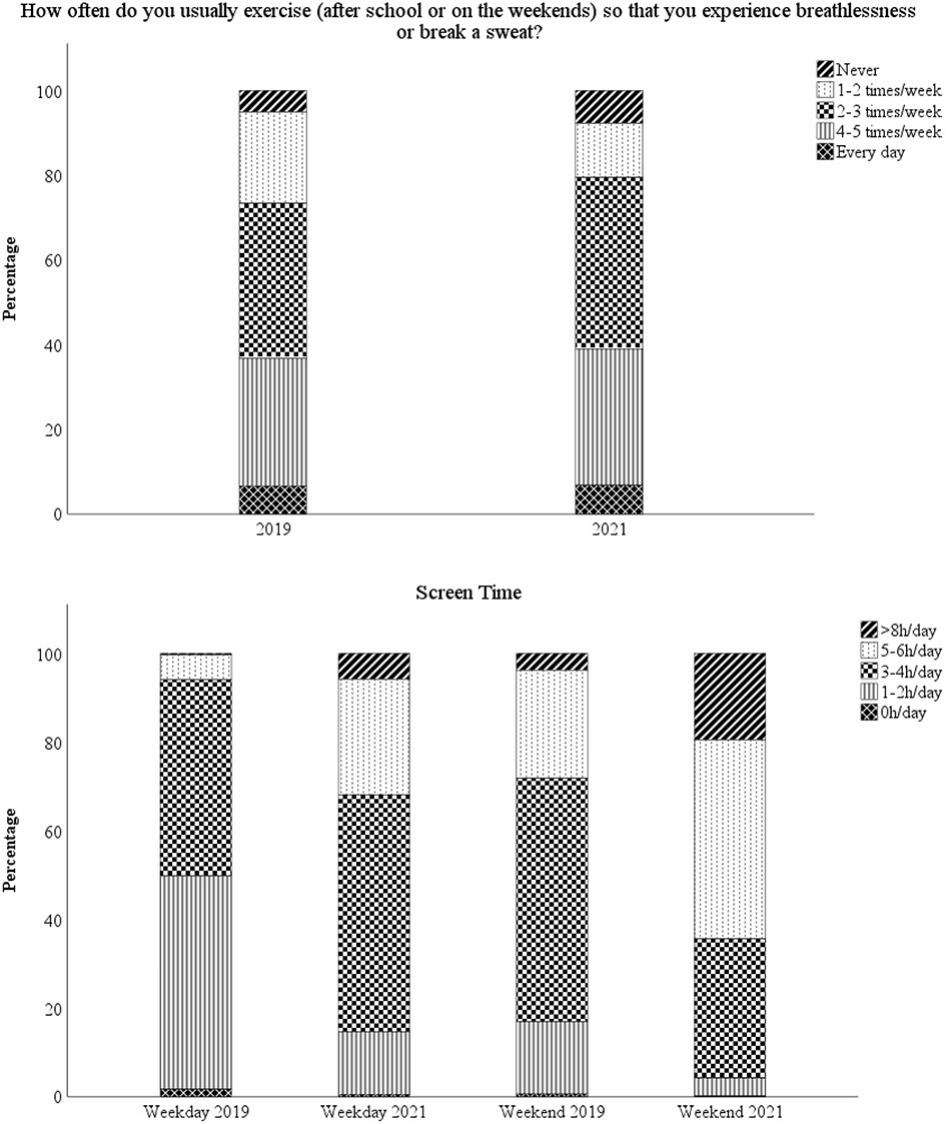
Graphs showing physical activity and screen time habits in 2019 and 2021 respectively, for children answering both in 2019 and 2021 (*n* = 777).

Weekly screen time in hours followed a normal distribution. The number of children who answered these questions were 875 in 2019 and 809 in 2021, with 758 answering at least one screen time question both years. For children answering both years, 11-year-old children reported using screens for 2.6 h on weekdays and 3.8 h at weekends making a total of 20.7 h a week (Table 3). By the age of 13, this had increased to 4.0 h on weekdays and 5.2 h at weekends, making a total of 30.1 h a week (Table 3). This was an increase of 9.4 h (95% CI 8.6–10.2 h) or 45.4% (95% CI 41.5–49.3%) (paired sample *t*-test *p* < 0.001) (Table 3 and Figure 2). The percentage who managed to meet the consensus goal of < 2 h of recreational screen time a day were 14.6% of 11-year-old children and 3.2% of 13-year-old children. This was a significant difference (*p* < 0.001) (Table 2). There was no difference in the overall mean screen time between the sexes at 11 (*p* = 0.781) and 13 (*p* = 0.966) years of age (data not shown), or by maternal educational level (Table 2).

**Table 3:** Screen time in hours per day and by category based on weekdays or weekends as well as for the entire week in 2019 and 2021 respectively.

**Screen time**	**Weekday 2019**	**Weekday 2021**	**Weekend 2019**	**Weekend 2021**	**Week 2019**	**Week 2021**	***p* ^a^**
Mean, h/day (SD)	2.60 (1.28)	3.96 (1.61)	3.81 (1.54)	5.16 (1.75)	20.65 (8.7)	30.11 (10.7)	**< 0.001** ^**b**^
Total^*^	722	758	722	758	722	758	
≤ 2 h/day, % (*n*)	49.8 (443)	14.7 (122)	16.9 (148)	4.2 (34)	14.5 (127)	3.0 (24)	**< 0.001** ^**c**^
3–4 h/day, %(n)	44.3 (394)	53.4 (442)	54.9 (480)	31.4 (254)	79.9 (699)	69.2 (560)	
≥5 h/day, % (*n*)	6.0 (53)	31.9 (264)	28.2 (247)	64.4 (521)	5.4 (47)	27.8 (225)	**< 0.001** ^**d**^
Total	100 (890)	100 (827)	100 (875)	100 (809)	875	809	707

^*^Only children answering both questionnaires in 2019 and 2021 with complete answers for screentime during both weekdays and weekends were included.

^a^Difference in weekly screen time, *p* < 0.05 regarded significant and marked in bold.

^b^Paired Sample *t*-test, *T* = −23.4.

^c^McNemar, Test Statistic 65.3.

^d^McNemar, Test Statistics 121.2.

The amount of screen time on weekdays and weekends followed the same patterns. High or low consumers on weekdays were high or low consumers at weekends. In 2019, we found that 53 children (6.0%) reported using screens for 5 h or more a day during the week, of these children 21 used screens for 5–6 h during the weekend as well, while 23 children increased their usage to more than 8 h a day at weekends, 4 children did not reply the weekend question and 5 children replied 3–4 h on the weekend (data not shown).

At the other end of the scale, none of the children who reported no screen use on weekdays reported using them for 5 h or more at the weekends and 64.3% used their screen for < 2 h a day at weekends. This was considerably lower than the 14.6% of all children who used them for < 2 h a day at weekends in 2019, Table 2.

In 2021, 809 replied to the question about screen time. Of these, 253 said they used screens for 5 or more hours per day during the week and 87.7% reported the same high usage at the weekends. A quarter (19.8%) of the 121 who reported < 2 h of screen time on weekdays at 13 years of age also used screens for < 2 h at the weekend and they accounted for 70.6% of the those with the lowest weekend usage (data not shown).

**Figure 3:**
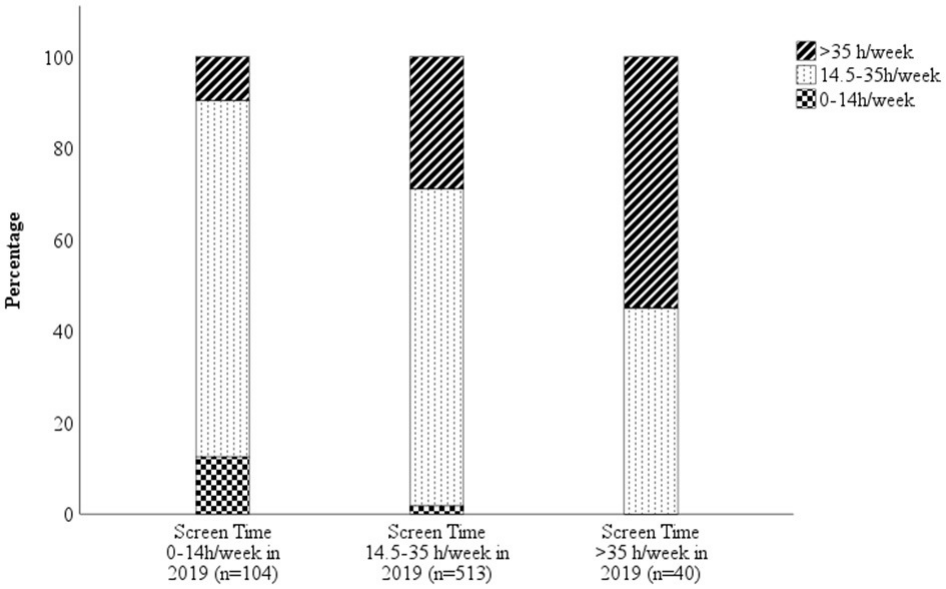
Amount of weekly screen time in 2021 based on the weekly screen time usage in 2019. The graph shows that patterns were established early, even though most children increased their screen time from 2019 to 2021. The graph also shows that of the 40 children who used screens for more than 35 hours per week in 2019 actually reported lower screen time in 2021, despite the ongoing pandemic.

Screen time habits were established early: 71.1% (*n* = 505) of the 707 children who replied to this question in both years increased their weekly screen time from 11 to 13 years of age, 9.8% reported a decrease and 19.1% reported no change (Figure 3). The RR for increase vs. unchanged screen time was 1.57, it was 1.76 for increased vs. decreased screen time and it was 1.32 for unchanged vs. decreased screen time. Of the 505 children who reported an increased screen time in 2021 compared to 2019, 49.3% did not think that they had used more screens the past year due to COVID-19. Comparative figures for the entire cohort were 68.5% who reported that they did not use more screens due to COVID-19.

### Links between PA and screen time before and during the pandemic

Adolescents who reported high screen time of at least 35 h per week at 11 years of age were more likely to say they never exercised than children with a low screen time of < 14 h a week (12.8% vs. 5%, *p* = 0.014). Those who reported low screen time engaged in physical activity more often than high screen users (51.2 % vs. 17.0% *p* < 0.001) and fewer high screen users were active in sports clubs than low screen users, (88.1% vs 66.7%, *p* < 0.001). At 13 years of age, 38.7% of low screen users and 26.3% of high screen users exercised at least 4–5 times a week (*p* = 0.044) (Table 4).

**Table 4:** Physical activity habits in 2019 and 2021 for the entire cohort and by high and low screen time.

		**Screen time in 2019**					**Screen time in 2021**			
	**Total, % (** ***n*** **)**	**Low: 0–14 h/week, % (** ***n*** **)**	**High:** >**35 h/week, % (*****n*****)**	**P** ^a^	**Effect size** ^b^	**Total, % (** ***n*** **)**	**Low: 0–14 h/week, % (** ***n*** **)**	**High:** >**35 h/week, % (*****n*****)**	**P** ^a^	**Effect size** ^b^
Children active in sports club	88.1 (690)	90.5 (105)	66.7 (30)	**< 0.001**	0.291	76.2 (615)	87.5 (21)	70.2 (158)	0.073	0.113
Children that reports no physical activity	5.0 (44)	3.1 (4)	12.8 (6)	**0.014**	0.185	7.7 (62)	0	13.4 (30)	0.091^c^	0.121
Sufficient PA^d^	36.1 (316)	51.2 (66)	17.0 (8)	**< 0.001**	0.306	38.7 (313)	45.8 (11)	26.3 (59)	**0.044**	0.128

^a^Difference calculated with the ChiSquare Test, *p* < 0.05 regarded significant and marked in bold.

^b^Effects size measured as Cramers V and interpreted as < 0.5 moderate < 0.3 small, < 0.1 negliable.

^c^Fischers exact test *n* < 5.

^d^Children that reported that they became breathless or broke a sweat at least 4–5 times/weekly in addition to mandatory physical education twice a week.

## Discussion

This was a comparative study of PA and screen use among 11-year-old Swedish children in 2019, before the pandemic, and the same children aged 13 during the pandemic in 2021. Most 13-year-olds exercised as frequently during COVID-19 as they did in 2019 and some even increased their frequency of PA. This was an unexpected finding, as it differed from what we expected due to their age and from what has been reported from other countries during the pandemic. On the other hand, screen time increased considerably for most Swedish children and this reflected global trends. These results are important, as Sweden was one of few countries that applied comparatively mild restrictions during the COVID-19 pandemic. This study highlights how different restrictions may influence changes in lifestyle habits, and therefore health, why these findings may be of international interest.

Our estimates suggest that ~40% of our Swedish cohort engaged in physical activity at least 4–5 times a week on their leisure time at the age of 11 and managed to maintain this level until 13 years of age, despite the pandemic. There were no differences between the sexes at the age of 11 and exercising at least 4–5 times a week but fewer girls reached this frequency compared to boys at the age of 13. For active school breaks, this difference between the sexes were seen already pre-pandemic at the age of 11 with fewer girls being active. On the other hand, more girls where active in sports clubs compared to boys in both 2019 and 2021. With the question formulation used, we may not conclude whether our estimate equals the WHO MVPA due to lack of time and intensity specification. Still, the question formulation specified that it was exercise that made the child become breathless or break a sweat which may be interpreted as at least moderate intensity physical activity. In addition, all children were offered mandatory physical education twice weekly. Trying to put this numbers into perspective, 40% of all children would thereby have been physically active at least 6–7 times a week during the pandemic. This was higher than the 16% expected to reach the WHO goal of 60 min of MVPA a day according to the global pre-pandemic meta-analysis from 2016 (9) and peri-pandemic studies from countries with extensive restrictions (18, 22, 24, 29, 30). However, our findings were similar to a German study (25) on young children and specific subpopulations of Belgian adults (26). Both these countries applied comparably mild restrictions, as schools and sports clubs were closed and social distancing rules applied, but outdoor activities were allowed. In contrast, countries such as China, Canada, the USA, Spain and Italy, had almost complete bans on sporting activities (18, 22, 24, 29, 30). The milder restrictions in Germany resulted in more time spent outdoors, which probably contributed to increased low to moderate intensity PA, but the general lockdown appeared to somewhat reduce MVPA. Sweden probably had the mildest restrictions of all of these countries, as children could still attend sports clubs and they maintained their frequency of exercise that Germany did not. In our cohort, 88.3% and 76.3% of children aged 11 and 13 were active in sports clubs in 2019 and 2021, respectively, and 97.9%, were able to continue their activities to some extent during the pandemic. Although, 63.5% reported that their PA routines had changed as a result of the pandemic, only 40.9% said this meant fewer exercise opportunities. Children who belonged to sport clubs were much less likely to report reduced PA during the pandemic than those who did not go to clubs (23.2% vs. 41.0%). This is important though children from lower socioeconomic backgrounds were less likely to belong to sports clubs in 2019 and this difference became larger during the pandemic, possibly increasing health gaps between different socioeconomic groups in society.

PA usually decreases with age (31, 32) and we expected to see a decrease in PA frequency without the pandemic. This positive finding could be explained by a number of factors. Sweden implemented social distancing and leisure activities were canceled, but outdoor activities and participation in sports clubs were maintained which might have been reason to continue PA participation in lack of other activities. Increased media attention on healthy behavior may also have helped.

Being able to attend school most of the time during the pandemic, maintained PA during school breaks for almost 10% of the children. Even so, this was a sharp decline as compared to the 29% of children who reported that they were often or always active during school breaks in 2019. School holidays has been associated with reduced PA and has been linked to the increased risk of obesity (33). It is therefore probable that keeping schools and sports clubs open, albeit with some social distancing restrictions, are two reasons why PA levels were maintained in Sweden during the COVID-19 pandemic.

When it came to screen time, 71.1% of all the children reported increased levels from 2019 to 2021, with an estimated mean increase from 20.7 h per week to 30.1 h per week, with no difference between the sexes. Increased screen time seems to have been a universal phenomenon during the COVID-19 pandemic (18, 24, 25), and we are not aware of any adverse findings being published. It is difficult to put these numbers into perspective, as screen time has been shown to increase with age (16, 34) and as the years have progressed (15, 34). We expected screen time to increase between 11 and 13 years of age, but suspect that the COVID-19 pandemic speeded up this evolvement. Despite this, 68.5% of children did not think that they had increased their screen time due to the pandemic. Why children responded this way we do not know, perhaps they increased their screen time as a result of increasing age since many families have screen time limits or perhaps, they were not aware that they had increased their screen time. Estimating the effect that the pandemic actually had on screen time is difficult, but our results corresponded rather well with the results of the 2021 Swedish PEP report. This is a yearly Internet-based questionnaire and in 2021 the link was sent out to a systematic probability selection of 29,000 children in Sweden aged 4–17 or their parents. The 2021 report stated that 30% of 8320 children aged 4–17 years reported increased screen time in 2021 compared to before the pandemic, because of canceled leisure activities and fewer opportunities to meet friends (15).

We were alarmed that only 14.5% of the 11-year-old pre-pandemic cohort adhered to the consensus guidelines of < 2 h of screen time per day (2, 3) and that this had fallen to 2.9% by 13 years of age. The numbers were slightly better when the data were split up into weekdays and weekends, but this level of screen use can have a negative impact on children's health. Swedish children appear to spend more time on screens at an earlier age than other countries. For example, a German study found that the percentage of children who used screens for < 2 h per days during the week and at weekends were 40.1% and 16.4% of those aged 11–13 years and 23.9% and 12.6% of those aged 14–17 years (25). A Spanish study of children aged 8–16 (30) reported that 66.0% used screens for at least 2 h per day before the pandemic and 87.7% during the pandemic (30). These results were similar to our study, but they may not be comparable as different age groups, definitions of screen time and questions were involved. Our aim was to describe differences in lifestyle habits over time and relate these to international results in the light of pandemic restrictions. Few children met the screen time recommendations globally, including Sweden, and screen time increased by 45.4% in just 2 years in our study even though Sweden had milder restrictions than many other countries. Part of this increase in screen time could have been due to limited socializing in person and canceled leisure activities during COVID-19. However, 68.5% of the children said that they had not used their screens more in 2021 because there was a pandemic. This means that increasing age and general time trends probably explain parts of the increase. We are concerned that this higher usage may not have decreased when restrictions were removed, but further studies are needed to see if that was the case.

When we looked at potential associations between PA and screen time, we found a negative correlation between high screen use of more than 5 h a day and a lower level of PA. For example, 17.0% of high screen users in 2019 reported exercising at least 4 times a week compared to 51.2% of children that used screens for < 2 h a day. At the other end of the scale, 12.8% and 3.1%, respectively, said they never exercised. This finding was in line with both a large meta-analysis by Carson et al. (5) that found that higher screen time was associated with lower fitness and with the PEP report (17). It was evident that screen time and PA did not have a linear relationship in our study, because the children spent twice as much time using screens as exercising. However, it was evident that high screen users exercised less than low screen users, especially at 11 year of age and/or pre-pandemic when screen time was generally lower.

Our study confirms the possibility that the pandemic contributed in part to reduced PA in some countries but may actually have increased PA in countries with mild restrictions due to the combination of restrictions applied. The approach that Sweden took has been questioned on transmission grounds, but maintaining opportunities for PA seems to have safeguarded healthy behaviors in our study cohort. We hope that this study will contribute to the knowledge on how lifestyle habits change over time and during restrictions. Exploring the impact that the COVID-19 pandemic has had on the lifestyle habits of children in different countries, may produce knowledge on future risks and benefits that national decisions may have on public health in possible future crises. These results may be seen as novel, but further studies are needed to confirm whether these findings can be generalized to other contexts.

### Strengths and limitations

A strength of this study was the longitudinal design, that enabled us to use the children's reports to compare individual changes in screen time and PA at two ages, before and during the pandemic. However, the generalizability of this population may be questioned, as we know that families with lower socioeconomic status tend to participate in studies less often than families with higher socioeconomic status (35). That was also seen in our study. The mothers who took part in this latest study had high educational levels, which was used as a proxy for socioeconomic level. This may have impacted the results, as children from families with lower socioeconomic status tend to be more affected by societal crises (15, 16). This socioeconomic effect on child PA was shown in our study. In 2019 there were no difference in PA by maternal education, except for the children's club membership. However, differences emerged during the pandemic with regard to all PA measures, albeit with a small effect size. This finding makes it probable that overall PA may be slightly lower in general than presented in this study. Despite this, our study had a large amount of respondents and maternal educational level and ethnic profiles were well in line with the PEP report (15), which is considered a well distributed scientific study. In 2020, that report stated that 78% of their respondents had a higher educational level, compared to the 43% quoted by Statistics Sweden, and that 19% of their respondents were of foreign origin, compared to 25% in Sweden (15). In addition, our study showed no differences between child-related factors and the general population of Halland. Despite these differences, which may affect generalizability, it is a strength of the study that mothers from other ethnic origins that Swedish were represented.

The limitations could have included recall bias, but our longitudinal approach reduced this potential error. There was also a risk for overestimating PA and underestimating screen time, due to self-reported data and social desirability. However, this would probably have affected the answers in 2019 and 2021 alike, because 777 of the 1,041 children who took part responded in both years. The effect of providing socially desirable answers may have been somewhat more apparent in 2021 due to increasing age. Another limitation that has already been mentioned was that the children were not asked to specify the exact time they spent on PA nor at what intensity why comparison toward the WHO guidelines is largely compromised. In addition, we only asked about exercise that made the child breathless or break a sweat why children may have been more active than usual but at lower than that intensity, as families engaged more in outdoor activities like visiting green areas during the pandemic (36). For example, low to moderate intensity PA increased in Germany (25) during the pandemic and this suggests that our cohort may have been even more active than our study suggests. Another questionnaire-related weakness was that we asked the children whether they did less exercise during COVID-19 and they were not asked about more PA. Furthermore, we did not ask them about how much screen time was for recreational use as we wanted comparable figures in 2019 and 2021. However, it is likely that most children interpreted the question as recreational use, as the same children had high screen time during the week and at weekends. In addition, with schools open, we anticipate that if screen time have been for homework, this might be the case for both years. These limitations may have affected the results in both ways and we believe that this study contributes useful and unique information. Further studies are needed to confirm the results and study the long-time-effects on screen time and PA.

## Conclusion

Most 13-year-old children living in the Halland region of Sweden maintained their frequency of PA during the COVID-19 pandemic and it was actually more common for them to report increased than decreased activity. This finding seems to be a unique phenomenon, when it is compared to studies from other countries during the COVID-19 pandemic. We believe that these findings were probably due to the fact that Sweden did not have the same high level of restrictions as other countries. Schools and sports clubs remained open, but there were some social distancing measures put in place. In more detail, girls were physically active less than boys and socioeconomic factors were negatively associated to physical activity during the pandemic. However, Swedish children did increase their screen time by 45.4% between 2019 and 2021 and most children exceeded the recommendations of 2 h per day. Part of this increase in screen time could have been due to COVID-19 measures, when leisure activities were canceled and children had limited opportunities to meet up with friends. Having said that, more than half of the children said that COVID-19 was not the reason for their increased screen usage and it is possible that their age and the fact that 2 years had elapsed may account for part of the increase. It is a concern that this higher usage may not have reduced after the restrictions were removed and further research is needed in this area.

## Data availability statement

The datasets presented in this article are not readily available because of data protection, but the appropriate authors may have access to and/or analyze the datasets of the current study if reasonably required. Requests to access the datasets should be directed to Josefine.Roswall@regionhalland.se.

## Ethics statement

The studies involving humans were approved by Etikprövningsnämnden, EPN Lund: 299/2007, EPN Lund: 141/2018 and EPM 02133/2021 Sweden. The studies were conducted in accordance with the local legislation and institutional requirements. Written informed consent for participation in this study was provided by the participants' legal guardians/next of kin. No potentially identifiable images or data are presented in this study.

## Author contributions

SB and JR conceived this study and were responsible for the data and interpretations. JR, GA-T, and SB were responsible for the questionnaires and study protocol. OW wrote the first draft and was responsible for the preliminary statistics before all the data were gathered as part of a master thesis. SB continued the work by reworking the statistics and writing the manuscript. All the authors read, revised and approved the final draft of the manuscript, and contributed with intellectual input. The corresponding author states that all the listed authors meet the authorship criteria and that no other authors that meet the criteria have been omitted.
