# Supplementary material for: Effects of the COVID-19 pandemic on the physical activity and screen time habits of children aged 11–13 years in Sweden
Source: Front Public Health. 2023 Aug 9;11:1241938. doi: 10.3389/fpubh.2023.1241938 (PMC10447604; doi:10.3389/fpubh.2023.1241938)
Supplement: Supplementary file 1 [file Data_Sheet_1.DOCX]

Supplementary Material

# Supplementary Appendix 1

**Measures
Physical activity and exercise habits**

***In 2019 the children were asked:***

- “Do you experience shortness of breath or break a sweat when you move/play during school breaks?” (yes, no)
- “How often do you usually exercise (after school or on the weekends) so that you experience breathlessness or break a sweat?” (every day, 4-5 times a week, 2-3 times a week, 1-2 times per week, never)

***In 2019 the parents were asked:***

- “Is your child physically active in a club or association outside of school?” (yes, no).

***In 2021 the children were asked:***

- “Do you experience shortness of breath or break a sweat when you move/play during school breaks?”.
- “Are you active in a sports-association/club or part of a team?”
- ”How often do you usually exercise (after school or on the weekends) so that you experience breathlessness or break a sweat?”
- “Have you been able to continue with your activities during the last year during the pandemic?”
- “Have your practices/activities been different during the last year due to COVID-19?” and “In what way did the practice/activity change: (pick one or more alternatives): “Activity / training canceled”, “Activity / training as usual but with fewer participants due to the restrictions", "Activity / training only outdoors" and "Fewer training opportunities”
- "Have you been less physically active (moved less) in the past year due to COVID-19?".

**Screen time habits**Screen time was estimated by these questions in both 2019 and 2021:

- “About how many hours (h) a day do you watch TV, use your phone, tablet or computer on a weekday?” and “About how many hours (h) a day do you watch TV, use your phone, tablet or computer on the weekend?” respectively. The alternatives were coded as follows: nothing: 0 hours, 1-2h: 1.5h, 3-4h: 3.5h, 5-6h: 5.5h, and 8h or more: 8h. To estimate a weekly screen time the answer for weekdays were multiplied by 5 and for weekends by 2. To reach the consensus goal of less than 2 hours a day a child had to reply either 0h or 1-2h both on weekdays and weekends.
- In addition, the following question was asked in 2021: “Do you experience that you have increased your screen time now during this year compared to before COVID-19?”.

**
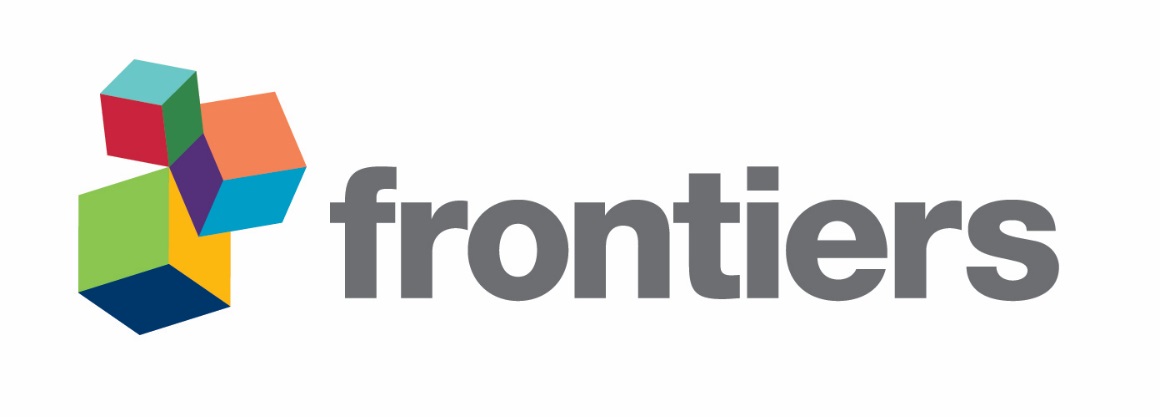
**
